# Could CT Radiomic Analysis of Benign Adrenal Incidentalomas Suggest the Need for Further Endocrinological Evaluation?

**DOI:** 10.3390/curroncol31090364

**Published:** 2024-08-25

**Authors:** Alessandro Toniolo, Elena Agostini, Filippo Ceccato, Irene Tizianel, Giulio Cabrelle, Amalia Lupi, Alessia Pepe, Cristina Campi, Emilio Quaia, Filippo Crimì

**Affiliations:** 1 Department of Medicine (DIMED), Institute of Radiology, University of Padova, 35122 Padua, Italy; toniolo.ales@gmail.com (A.T.); elena.agostini.1@studenti.unipd.it (E.A.); giulio.cabrelle@gmail.com (G.C.); amalia.lupi@phd.unipd.it (A.L.); alessia.pepe@unipd.it (A.P.); emilio.quaia@unipd.it (E.Q.); 2 Endocrinology Unit, Department of Medicine (DIMED), University of Padova, 35122 Padua, Italy; filippo.ceccato@unipd.it (F.C.); irene.tizianel@gmail.com (I.T.); 3 Department of Mathematics (DIMA), University of Genova, 16126 Genoa, Italy; cristina.campi@unige.it

**Keywords:** radiomics, machine learning, adrenals, incidentaloma, adenoma, hormonal hypersecretion

## Abstract

We studied the application of CT texture analysis in adrenal incidentalomas with baseline characteristics of benignity that are highly suggestive of adenoma to find whether there is a correlation between the extracted features and clinical data. Patients with hormonal hypersecretion may require medical attention, even if it does not cause any symptoms. A total of 206 patients affected by adrenal incidentaloma were retrospectively enrolled and divided into non-functioning adrenal adenomas (NFAIs, n = 115) and mild autonomous cortisol secretion (MACS, n = 91). A total of 136 texture parameters were extracted in the unenhanced phase for each volume of interest (VOI). Random Forest was used in the training and validation cohorts to test the accuracy of CT textural features and cortisol-related comorbidities in identifying MACS patients. Twelve parameters were retained in the Random Forest radiomic model, and in the validation cohort, a high specificity (81%) and positive predictive value (74%) were achieved. Notably, if the clinical data were added to the model, the results did not differ. Radiomic analysis of adrenal incidentalomas, in unenhanced CT scans, could screen with a good specificity those patients who will need a further endocrinological evaluation for mild autonomous cortisol secretion, regardless of the clinical information about the cortisol-related comorbidities.

## 1. Introduction

Endogenous hypercortisolism is a rare disease, with an incidence from 2 to 3 per million people annually [1]. On the contrary, mild autonomous cortisol secretion (MACS) detected in up to one-third of patients with an adrenal incidentaloma is not an uncommon condition [2] because up to >10% of patients aged 70 or over can present an incidentally detected adrenal mass [3]. Cardiometabolic morbidities (diabetes, hypertension, dyslipidemia) and mortality are increased in patients with MACS compared to those with a non-functioning adrenal adenoma [4]. Adrenocortical adenomas (ACAs) or adrenal adenomas are benign neoplasms that originate from the adrenal cortex [5]. Therefore, since non-functional adenomas and those with mild hormonal secretion may not produce noticeable symptoms and can remain asymptomatic for years, it is important to detect those with MACS as soon as possible in order to consider a proactive management aiming to reduce new-onset cardiovascular complications for the patient. The evaluation of an adrenal incidentaloma requires a comprehensive approach involving imaging and hormonal workup.

ACAs frequently contain a large amount of intracytoplasmic fat, which allows quantitative analysis with attenuation measurements at unenhanced CT. On the basis of their review of previously published series combined with their own results, Korobkin et al., using a threshold of 10 HUs, found an overall sensitivity of 73% and specificity of 96%. Therefore, they concluded that further follow-up is unnecessary when the attenuation of the lesion measures 10 HUs or less. The threshold has been also confirmed in the 2023 update of the European Guidelines [2,6,7]. The guidelines consider that no further evaluation is suggested in benign adrenal incidentalomas with low attenuation value, homogeneous texture, and diameter <4 cm [2]. However, ACAs’ behavior can change over time, with an increased cardiovascular risk [8]. We observed a modification in the attenuation value (and, therefore, lipid content in the ACAs) in patients that will develop MACS in the follow-up [9], underlining that there is a possible connection between imaging features of the adenoma and their secretory profile. Few radiomics and machine learning studies on the topic of adrenal glands and hormone secretion are available, but they are mainly studies on aldosterone secretion or comparison of adenoma subtypes [10,11,12].

The aim of our study was to test the possibility of differentiating not functioning adrenal incidentalomas (NFAIs) and adrenal nodules with MACS on the basis of clinical and radiomics features extracted from CT images performed for reasons other than the study of adrenal glands, even in the setting of an Emergency Department. This possibility would suggest the usefulness of developing additional predictive models in understanding when to refer a patient for endocrinologic evaluation for the possible presence of MACS.

## 2. Materials and Methods

### 2.1. Patients

This retrospective study was approved by the Ethics Committee of Padova University Hospital (protocol number 53401-2021). Between 2005 and 2020, we retrospectively enrolled all patients who had incidental CT findings of benign adrenal incidentaloma (adrenal nodule with mean densitometry at CT unenhanced scan <10 HUs or with densitometry between 10 and 20 HUs that showed, with MRI chemical shift imaging, a significant signal drop on opposed-phase images) who were referred to the endocrinology department at the University of Padova. Inclusion criteria were the following: (1) be evaluated at our third-level referral hospital and have undergone adrenal CT, including an unenhanced scan, with a 3 mm slice thickness or less, and (2) have a complete biochemical panel of hormonal secretion and availability of clinical data.

Clinical data collected included gender, age, body weight, and height (to calculate BMI), as well as the presence of cortisol-related comorbidities, such as hypertension (systolic or diastolic blood pressure >130/90 mmHg or antihypertensive treatment), diabetes mellitus (increased fasting blood glucose or HbA1c, antidiabetic treatment), dyslipidemia, and osteoporosis (lumbar or femoral t score <−2.5 or clinical evidence of frailty fractures). MACS was defined in the case of morning serum cortisol >50 nmol/L after a 1 mg dexamethasone suppression test.

**Figure 1:**
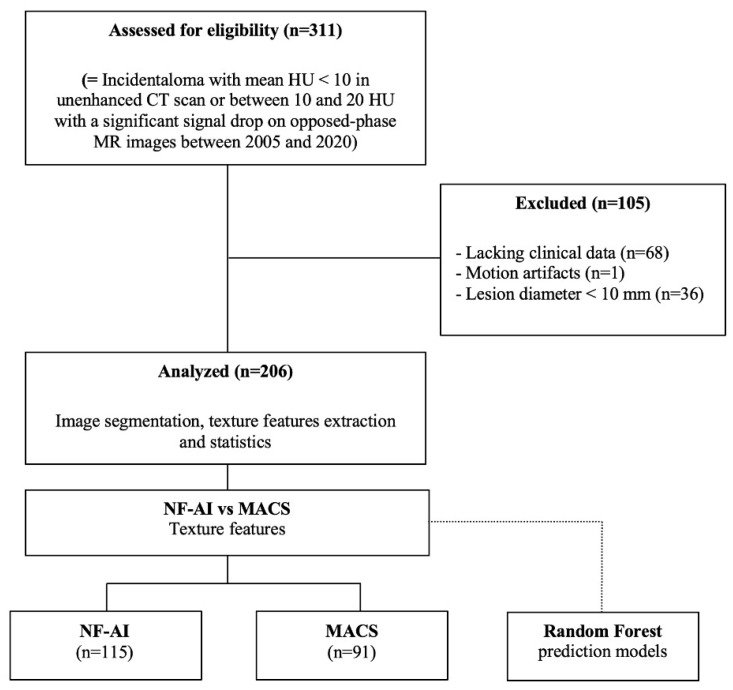
Flowchart of the study population accrual with inclusion and exclusion criteria and sample sizes.

The exclusion criteria were (1) axial maximum diameter of the lesion <10 mm; (2) poor-quality CT images; and (3) motion artifacts. A total of 206 patients (115 classified as not functional adrenal incidentalomas [NFAIs] and 91 as MACS) were finally selected for image analysis (Figure 1).

### 2.2. Imaging Protocol

The CT scanner used in the period between 2005 and 2020 at our institution was a 64-slice CT scanner (Somatom Sensation, Siemens Healthineers, Erlangen, Germany) with the following parameters: craniocaudal image acquisition with a 120 kV tube voltage, 250 mAs effective dose, 0.5 s rotation time, 0.6 mm detector collimation, and 0.75 pitch. The slice thickness for unenhanced scans was 3 mm, and the reconstruction kernel was 30B. All CT scanners were calibrated every morning before the first patient, according to the University Hospital of Padova standard procedures, and were maintained according to the manufacturer’s specifications.

### 2.3. Radiomic-Based Machine Learning Modeling

Each CT scan was retrieved from the institutional archive system, anonymized, and loaded on a dedicated workstation, where it was analyzed with an independently developed open-access image analysis software for texture analysis (LIFEx, Local Image Features Extraction, Orsay, France) [13]. All CT images were resampled to a voxel size of 1 × 1 × 3 mm (X spacing, Y spacing, Z spacing). Two abdominal radiologists (5 and 10 years of experience) blinded to clinical and histopathological data identified the adrenal nodule; in the case of bilateral nodules, the biggest one was selected, and a region of interest (ROI) was manually drawn along the tumor margins in each axial slice of unenhanced scans.

**Figure 2:**
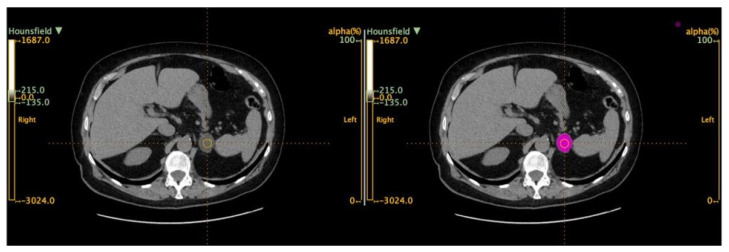
Example of segmentation (ROI) of a left adrenal incidentaloma.

A volume of interest (VOI) for each tumor was hence obtained (Figure 2).

LIFEx software (v. 7.3.0) was used to analyze the voxels within the entire VOIs and compute a set of textural parameters for each of them. Discretization was performed with a number of gray levels of 400 and a bin size value of 10, and rescaling was set to absolute values, with a minimum bound of −1000 and a maximum bound of 3000 HU. A total of 136 radiomic features were extracted from the densitometry data of both the first and second order. First-order statistics describe the distribution of pixels in the VOI using histograms, whereas second-order statistics describe how many neighboring pixels have the same gray level and their relationship in the image.

### 2.4. Statistical Analysis

Data are expressed in percentage, mean ± standard deviation (SD), or median and inter-quartile range (IQR), as appropriate. The Shapiro–Wilk test was used to test the normality of the parameters. For the comparison of clinical data, Student’s *t*-test or the Mann–Whitney test, as appropriate, was used for quantitative variables, and the chi-square test was used for categorical variables.

The adrenal nodules were divided into NF-AI and MACS on the basis of the cortisol secretion identified at biochemical examination. In order to remove highly correlated features, correlation coefficients for each feature were calculated, and those with a coefficient >0.6 or <−0.6 were excluded from the following analyses.

The entire cohort was randomly divided into a training group of roughly 2/3 of the patients and a validation cohort of 1/3 of the patients. The balance between MACS and NF-AI classes in the training cohort reflects the balance in the whole dataset (around 40%). Random Forest was used both in the training and validation cohorts to test the accuracy of CT textural features and cortisol-related comorbidities in identifying MACS patients. The level of significance was set to *p* < 0.01. Statistical analysis was performed using R statistical software (version 2.14.0; R Foundation for Statistical Computing, Vienna, Austria) [14].

## 3. Results

Two hundred and six patients with incidentally discovered adrenal incidentalomas were retrospectively enrolled for this study. The patients were, respectively, 45% (n = 93) male and 55% (113) female, with a mean age of 65 years (standard deviation [SD] ±9 years). Out of the whole cohort, 133 patients (65%) had high blood pressure, 38 (18%) diabetes, 84 (41%) dyslipidemia, and 22 (11%) osteoporosis.

On the basis of endocrinological evaluation and biochemical data, the patients were categorized, respectively, as 115 NFAI and 91 MACS. The MACS group compared to the NFAI one was significantly older at diagnosis (67.2 ± 8.7 years vs. 64.0 ± 9.8 years; *p* = 0.009) and showed lower basal ACTH levels (14.1 ± 9.9 ng/L vs. 18.6 ± 11.2 ng/L; *p* = 0.003), a higher mean diameter of the largest adrenal nodule at CT (22.7 ± 7.3 mm vs. 18.1 ± 6.1 mm; *p* < 0.001), and a higher percentage of patients affected by osteoporosis 16% vs. 6%; *p* = 0.015). The clinical data and the presence of NF-AI and MACS in the two groups did not differ significantly (Table 1).

The entire group of 206 patients was randomly divided into a training cohort of 143 patients and in a validation cohort of 63 (Table 2).

From the entire set of 136 textural features extracted from CT images, after the calculation of the correlation coefficients, 12 parameters were selected for the following part of the analysis. The feature selection process was performed based on correlation or anti-correlation values, excluding those with a coefficient greater than +0.6 (60%) or less than -0.6. The feature showing the highest number of correlations was retained for each selection. The correlation plot with the correlation coefficients calculated for each feature and their respective relationships is shown in Supplemental Figure S1 and Supplemental Table S1.

**Figure 3:**
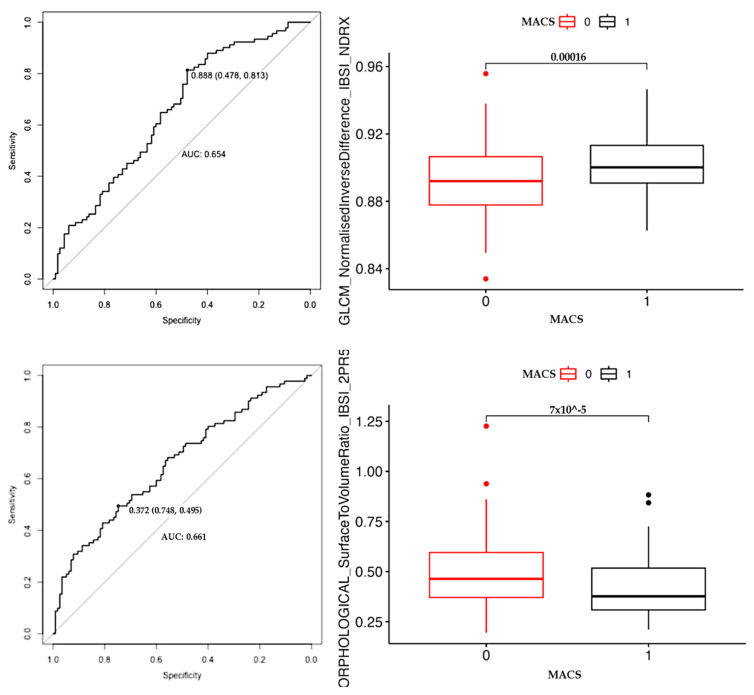
Results of correlation analysis (Wilcoxon test) and ROC curves on the direct comparison between the NF-AI and MACS groups of two of the most important features subsequently used to develop the Random Forest model.

The meaning of each parameter selected is reported in Table 3. Both first- and second-order radiomic features were selected for model development in addition to dimensional indicators. Some of the most important features employed in the predictive model showed correlations with very high statistical significance in the comparison between the NF-AI and MACS groups, e.g., “MORPHOLOGICAL Surface to Volume Ratio” and “GLCM Normalised Inverse Difference” demonstrated in the Wilcoxon test a *p*-value < 0.01 (Figure 3). Further explanation of their meaning is provided in Table 3.

**Figure 4:**
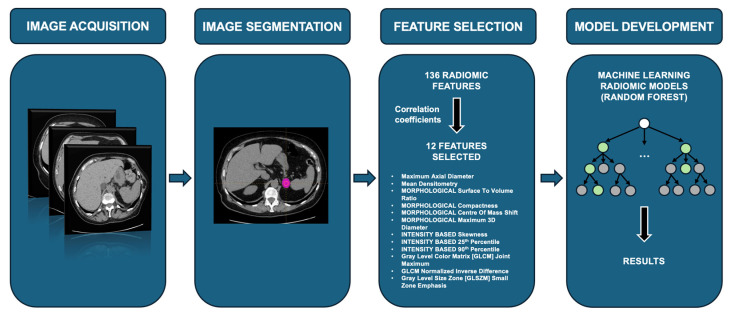
Conceptual steps of the study from image acquisition to predictive ML model development.

In the training group, the Random Forest correctly classified 100% of MACS and NF-AI, with 100% sensitivity and specificity (Figure 4). In the validation cohort, the sensitivity was 39% (95% confidence interval [CI]: 23–57%), the specificity 81% (95% CI: 62–94%), the negative predictive value 50% (95% CI: 35–65%), and the positive predictive value 74% (95% CI: 49–91%). Interestingly, if data on the presence or absence of hypertension, diabetes, dyslipidemia, and osteoporosis were added to the model, the results did not differ, confirming a sensitivity and specificity of 100% in the training cohort and a sensitivity of 39% and a specificity of 81% in the validation group (Table 4).

## 4. Discussion

In our study, an unenhanced CT-based radiomics model was shown to be able to distinguish between MACS and NF-AI patients with good specificity (81%), showing an acceptable positive predictive value (74%). The moderate–low sensitivity and moderate negative predictive value may be acceptable since radiomic analysis is available at no cost, and a false-negative patient with any other clinical suspicion of endocrine dysfunction would be anyway referred for harmless endocrinological screening. Furthermore, the prevalence of MACS in the general population is relatively low, as the prevalence of adrenal incidentalomas in the adult age is about 5%, and it has been found that only 9% of adrenal incidentalomas are mildly secreted [16,17]. For the “rule of thumb”, it is often presupposed that individuals with negative results can be “ruled out” if the screening test is highly sensitive and “ruled in” if the screening test is highly specific [18]. In an article by Power et al. [19], it is illustrated how tests with high specificity and low sensitivity are useful in ruling diagnoses in because if the prevalence of the condition is very low (as it is with screening), a test has to be very highly specific to reduce the number of false-positive results to an acceptable level. Furthermore, the results did not change, even by adding clinical information on comorbidities linked to excess cortisol secretion, such as hypertension, diabetes, dyslipidemia, and osteoporosis.

These findings suggest that a radiomics analysis of non-contrast CT images, even in an emergency setting not dedicated to the study of the adrenal glands, may be able to differentiate patients with mild cortisol hypersecretion who may benefit from endocrinological evaluation from patients with a non-functioning adrenal adenoma.

One of the jobs of a radiologist examining a CT scan is to identify adrenal nodules and tell treating doctors whether they are benign or malignant. The cut-off of 10HU as a mean densitometry to distinguish between a benign adrenal lesion and an adrenal mass suspected of being malignant is consolidated in the literature [2]. On the other hand, there are no radiological criteria to suspect that an adrenal nodule may be hypersecretory. Obviously, it is simpler and easier to perform a biochemical examination, but often after the identification of a benign adrenal nodule, the endocrinological/biochemical evaluation could be skipped for various reasons. The main one is that both the radiologist who identifies the nodules and the general practitioner who treats the patient are normally satisfied with a diagnosis of a benign lesion of the adrenal glands, thinking that no other tests are necessary.

It has been reported in the literature that in a case of recognition of adrenal nodules during a CT examination, even performed at a reference university hospital, for reasons other than the study of the adrenal glands, only a few of these patients are referred to the endocrinologist and to further biochemical evaluation, surprisingly even in case of clinical comorbidities directly linked to hormonal hypersecretion. For example, in the study by Kirsch MJ et al. [20] of 6913 patients who underwent CT colonography between 2004 and 2012, 148 patients had an adrenal incidentaloma. Among these, only 6.4% had a complete workup and 8% had a cortisol evaluation. Interestingly, even among patients who had a comorbidity related to cortisol hypersecretion, such as hypertension, diabetes, and osteoporosis, only 11.3% underwent biochemical testing for cortisol. The possibility of using a radiomics tool that can directly detect from CT images whether or not patients have cortisol hypersecretion could simplify the workflow and allow patients to be referred correctly.

The long-term deteriorating effects of cortisol hypersecretion, even in the case of MACS, are well known [4], and recently, there has been increasing attention towards MACS since it has been shown that a subclinical hypersecretion and an excess of cortisol for years could, however, lead to cortisol-related comorbidities and, therefore, to a worsening of the patient’s clinical prognosis.

Some limitations should be acknowledged in our study. First of all, it is a single-center retrospective study; although the cohort analyzed has a good number of patients divided into a training cohort and a validation cohort, the exams were acquired with the same CT scan and with the same protocol. Second, having sample sizes and data linked to only one center tends to bring out the problem of data overfitting. In machine learning, overfitting occurs when an algorithm fits too closely or even exactly to its training data. Different configurations of the model were attempted with the available parameters, but the phenomenon could not be completely eliminated in the training group; however, good specificity and positive predictive values were still obtained in the validation and test set, suggesting that the model may be able to generalize to new data. In addition, segmentation of adrenal nodules was manual and, therefore, even when performed by two radiologists in consensus, could be prone to errors. We preferred to choose a manual segmentation method of the adrenal nodules because of the risk of including healthy parts of the adrenal glands in the analysis, even if, in the literature, effective automatic tools have been validated for this purpose [21,22].

Finally, to be applicable on a large scale, the results should be validated in a multicenter cohort, even with different CT scanners.

## 5. Conclusions

Our study showed the possible usefulness of CT radiomics and machine learning in the context of a newly diagnosed adrenal incidentaloma with benign features in unenhanced CT scans, developing a predictive model that attempts to recognize adrenal nodules that are at risk of presenting MACS, with good specificity and PPV. Further studies and validation on this type of algorithm are needed, and prospective studies are necessary to generalize our findings; however, this represents a good starting point for the future, as the model can suggest whether to proceed with further biochemical and endocrinological evaluation in the general patient population.

## Figures and Tables

**Table 1:** Clinical data comparison between the NFAI and MACS groups.

	NFAI (n = 115)	MACS (n = 91)	*p*-Value
Age at diagnosis (years)	64.0 ± 9.8	67.2 ± 8.7	0.009 *
Gender, female/male (% female)	59/56 (51%)	54/37 (60%)	0.232
BMI (kg/m^2^)	30.4 ± 5.0	28.0 ± 4.5	0.578
Basal ACTH (ng/L)	18.6 ± 11.2	14.1 ± 9.9	0.003 *
HbA1c (mmol/mol)	41.4 ± 8.0	42.5 ± 9.6	0.422
Mean diameter (mm)	18.1 ± 6.1	22.7 ± 7.3	<0.001 *
Mean attenuation value (HU^m^)	−1.0 ± 10.1	1.8 ± 11.6	0.103
Hypertension (%)	70 (60%)	63 (68%)	0.036 *
Diabetes mellitus (%)	20 (17%)	18 (20%)	0.191
Dyslipidemia (%)	47 (40%)	37 (40%)	0.943
Osteoporosis (%)	7 (6%)	15 (16%)	0.015 *

NFAI, non-functioning adrenal incidentaloma; MACS, mild autonomous cortisol secretion; HU^m^, mean of the Hounsfield Unit in unenhanced CT; UFC, urinary free cortisol; BMI, body mass index; HbA1c, glycated hemoglobin; * = *p* < 0.05.

**Table 2:** Clinical data comparison between the training and test set selected to train the ML model. Proportions and rates were calculated for categorical data.

	Training Set (n = 143)	Test Set (n = 63)	*p*-Value
Age at diagnosis (years)	64.5 ± 9.8	67.7 ± 8.1	0.238
Gender, female/male (% female)	73/70 (51%)	41/22 (65%)	0.068
Number of incidentalomas	1.1 ± 0.3	1.1 ± 0.3	0.881
Fasting blood glucose (mg/dL)	107 ± 22.0	104.3 ± 23.2	0.704
Oncologic history (%)	33 (23%)	11 (17%)	0.436
Hypertension (%)	110 (77%)	51 (81%)	0.467
Diabetes mellitus (%)	25 (17%)	13 (21%)	0.576
Dyslipidemia (%)	69 (48%)	32 (51%)	0.703
Osteoporosis (%)	12 (8%)	7 (11%)	0.766

Continuous data were reported as means and standard deviation (SD).

**Table 3:** Explanation of each parameter selected after ICC analysis for the development of the machine learning model [15].

Parameter Name	Meaning
Maximum_axial_diameter	Maximum 2D dimension in the axial plane of the incidentaloma.
Mean_densitometry	Mean densitometry of the adenoma in the HU.
MORPHOLOGICAL Surface_to_Volume_Ratio	The ratio between the surface area and volume of an object; lower values mean that the incidentaloma tends toward a spherical shape, in contrast to elongated or heterogeneous shapes.
MORPHOLOGICAL Compactness 1	The compacity feature reflects how compact the volume of interest is. Compacity = A3/2V, where V and A correspond to the volume and the surface of the volume of interest based on the Delaunay triangulation.
MORPHOLOGICAL Centre_Of_Mass_Shift	Distance in millimeters between the normalized sphere radius of the activity hotspot with a weighted center of mass.
MORPHOLOGICAL Maximum_3D_Diameter	Maximum dimension in every plane of the incidentaloma.
INTENSITY_BASED Skewness	Measures the asymmetry of the distribution of values about the mean value. Depending on where the tail is elongated and the mass of the distribution is concentrated, this value can be positive or negative.
INTENSITY_BASED 25%_Percentile	Density value below which 25% of the image pixel density values are located (first quartile).
INTENSITY_BASED 90th_Percentile	Density value below which 90% of the image pixel density values are located.
GLCM Joint_Maximum	Gray Level Co-Occurrence Matrix (second order feature) Joint Maximum—in other software called “maximum probability”—measures the largest probability of occurrence of a specific gray-level value in the GLCM matrix. It is calculated by finding the maximum value in the GLCM matrix.
GLCM Normalised_Inverse_Difference	Measure of the local homogeneity of an image.
GLSZM Small_Zone_Emphasis	Gray Level Size Zone Matrix (second order feature) Small Zone Emphasis—measures the distribution of small size zones.

**Table 4:** Radiomics model performance on the training and validation cohort.

Training Cohort	Biochemically Confirmed MACS	Biochemically Confirmed NF-AI	Total
Radiomics model classification as MACS	55	0	55
Radiomics model classification as NF-AI	0	88	88
	55	88	143
Apparent prevalence	38%		
True prevalence	38%		
Sensitivity	100%		
Specificity	100%		
Positive predictive value	100%		
Negative predictive value	100%		
**Validation cohort**	**Biochemically confirmed MACS**	**Biochemically confirmed NF-AI**	**Total**
Radiomics model classification as MACS	14	5	19
Radiomics model classification as NF-AI	22	22	44
	36	27	63
Apparent prevalence	30%		
True prevalence	57%		
Sensitivity	39%		
Specificity	81%		
Positive predictive value	74%		
Negative predictive value	50%		

## Data Availability

The data presented in this study are available upon request from the corresponding author.

## Supplementary Materials

The following supporting information can be downloaded at https://www.mdpi.com/article/10.3390/curroncol31090364/s1, Figure S1: correlation plot of the different radiomic features after calculation of the respective coefficients; Table S1: values of correlation coefficients calculated on each radiomic feature.
