# Supplementary figures and images for: Could CT Radiomic Analysis of Benign Adrenal Incidentalomas Suggest the Need for Further Endocrinological Evaluation?
Source: Curr Oncol. 2024 Aug 25;31(9):4917–26. doi: 10.3390/curroncol31090364 (PMC11431504; doi:10.3390/curroncol31090364)

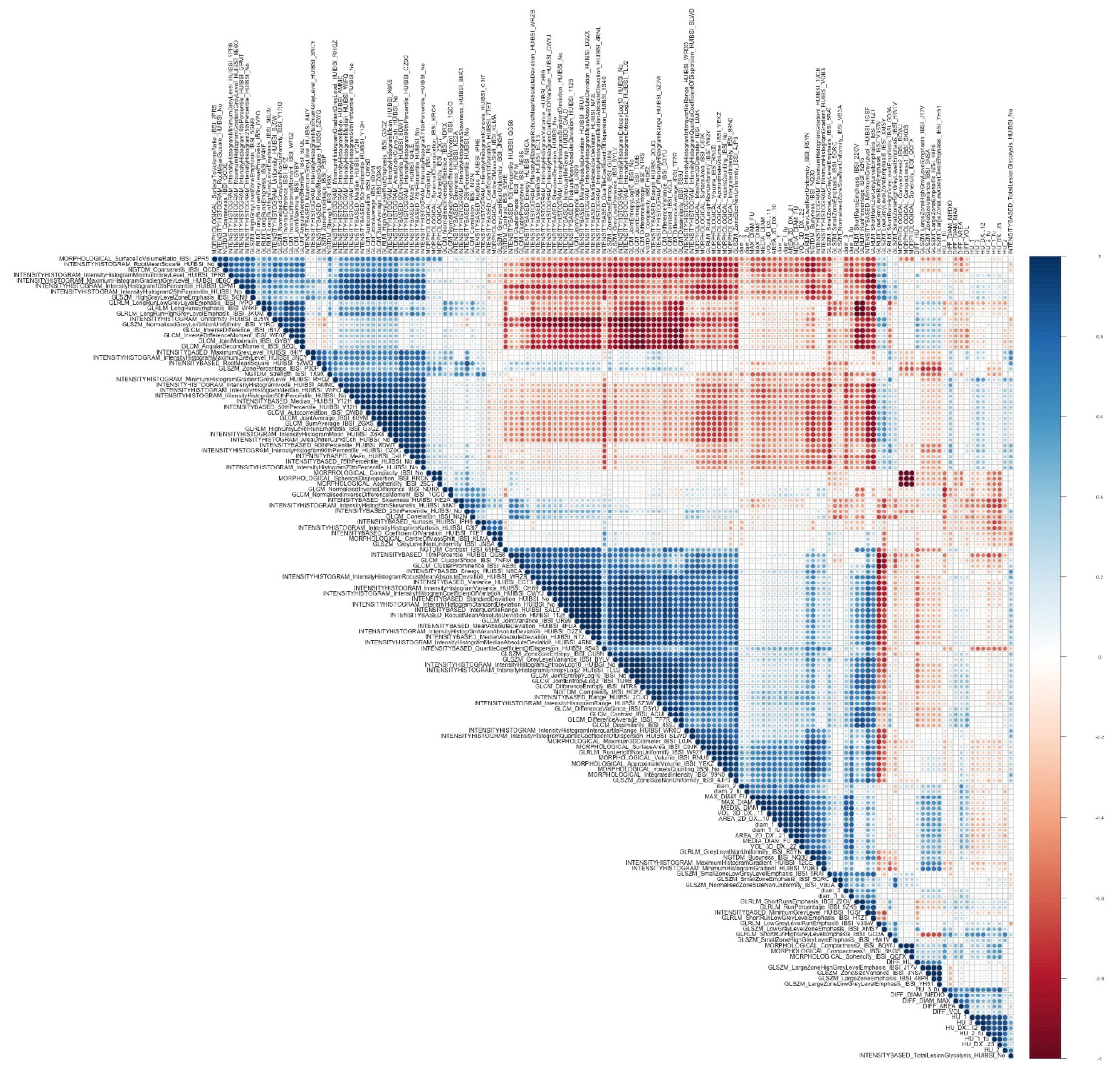

Supplement: Supplementary file 1 [file curroncol-31-00364-s001.zip › Supplemental Figure S1.tiff]
